# The effect of phacoemulsification plus goniosynechialysis in acute and chronic angle closure patients with extensive goniosynechiae

**DOI:** 10.1186/s12886-019-1070-9

**Published:** 2019-03-04

**Authors:** Tian Tian, Mei Li, Yingzi Pan, Yu Cai, Yuan Fang

**Affiliations:** Department of Ophthalmology, Peking University First Hospital, 8 Xi Shi Ku Street, Xi Cheng District, Beijing, 100034 People’s Republic of China

**Keywords:** Goniosynechialysis, Phacoemulsification, Angle closure, Cataract

## Abstract

**Background:**

Patients with primary angle closure/glaucoma (PAC/PACG) with extensive peripheral anterior synechiae (PAS), and coexisting cataract, increasingly have been treated with phacoemulsification combined with goniosynechialysis (Phaco-GSL). Since the mechanisms of acute and chronic PAC/PACG may differ, the treatment effect of this procedure also may differ. The purpose of this study was to establish whether there was a difference in the therapeutic effect of Phaco-GSL on these two groups of patients, the results of which could provide clinical evidence for improvement in treatment protocols for patients with PAC/PACG and extensive PAS.

**Methods:**

This study was a retrospective cohort study. Twenty-seven patients, 13 with acute PAC/PACG and 14 with chronic PAC/PACG, were treated surgically by Phaco-GSL. The intraocular pressure (IOP), surgical success rate, the need of medication, the extent of PAS, the time and the rate of recurrence of PAS (re-PAS) and other indicators were observed post-operatively for at least 3 months.

**Results:**

After surgery, IOP decreased (preoperative vs postoperative: 29.77 ± 11.55 mmHg vs 14.92 ± 1.66 mmHg in the acute group and 26.00 ± 11.2 mmHg vs 14.93 ± 2.7 mmHg in the chronic group), the extent of PAS reduced (preoperative vs. postoperative: 314.23 ± 49.07° vs 116.54 ± 73.78° in the acute group and 285.00 ± 53.28° vs 156.43 ± 56.35° in the chronic group), the topical and systemic anti-glaucoma drug requirements decreased, in both groups and in the acute group, respectively. Compared with the acute group, the success rate (acute vs chronic: 100% vs 64.3%) was lower in the chronic group, while the incidence of re-PAS (acute vs chronic: 30% vs 83.3%) were higher in the chronic group. All differences mentioned above were statistically significant (*p* < 0.05). In addition, there were five patients in total who showed re-PAS of more than 90° (4 in chronic group and 1 in acute group) and all these re-PASs formed within 1 week postoperatively.

**Conclusion:**

Although Phaco-GSL is effective in both groups, there may be differences in the effect between the two groups. Chronic patients are more susceptible to re-PAS. Thus, these patients should be observed closely and treated appropriately in the early post-surgical time period.

## Background

PAC and PACG are highly prevalent in Asians, especially in East Asians [1]. In China, 38.3% of PACG patients suffer from unilateral or bilateral blindness [2]. When the degree of PAS is limited, laser peripheral iridotomy (LPI) alone, or combined with laser iridoplasty is the preferred method of treatment for PAC/PACG patients, while trabeculectomy is traditionally employed to treat patients with extensive PAS. Complications such as shallow anterior chamber, cataract progression, filtering bleb scarring, endophthalmitis and other risks, exist for trabeculectomy [3]. Phacoemulsification plus Phaco-GSL, a surgical procedure in which the shallow anterior chamber is deepened and the adhered angle is re-opened in an attempt to restore trabecular function, is an alternative procedure which has been shown to successfully lower the IOP in PAC/PACG patients with coexisting cataract. In addition to having a very similar IOP lowering effect, Phaco-GSL has certain advantages over trabeculectomy, especially when considering the possibility of complications [4–8].

In addition to re-opening an adhered angle, the primary effect of Phaco-GSL is relief of lens-related pupillary blockage. However, there are other non-pupil blockage mechanisms involved with the development of PAC/PACG. Yan YJ and coauthors [9] observed PAC/PACG patients after LPI, a procedure to relieve pupil blockage, and found that approximately 29.1% (39/134) of the eyes which had undergone the procedure, retained at least two quadrants of appositional angle closure. They found several factors causing angle closure after LPI, including anterior position of the iris, thick peripheral iris, and plateau iris. Previous studies have shown that acute PAC/PACG is caused predominantly by pupillary block, while chronic PAC/PACG often involves other factors in addition to pupillary block [10]. Since not as many factors contribute to angle closure in acute PAC/PACG patients, Phaco-GSL might be more effective in these patients, than in chronic patients, for whom, in some cases, the procedure did not completely relieve the closure. Thus, the risk of angle re-attachment in chronic PAC/PACG patients was higher than for those in the acute group, which could have affected the surgical outcome. White AJ and coauthors [11] studied PAC/PACG patients and compared the effect of Phaco+GSL in the two groups, those with and those without an acute episode. It was found that the IOP in those with an acute episode decreased more significantly after surgery than those without an acute attack. The authors proposed that the reason for this difference was that the pre-operative IOP in the acute group was higher than that in the chronic group. Their results also showed that there was no significant difference in IOP, or in the number of drugs used between the two groups post-surgically. But they did not compare other aspects of the surgery between the groups, such as treatment success rate and the conditions for re-PAS. In addition, their inclusion criteria for patient did not specify the range of PAS. Until now there has not been much discussion on these points.

The purpose of our study was to analyze the effect of Phaco-GSL in the treatment of acute and chronic PAC/PACG patients with extensive PAS and concomitant cataract. Besides the effect of lowering IOP, in order to provide further clinical data for treatment of these patients, we also analyzed the incidence, the time and the extent of the re-PAS postoperatively.

## Methods

### Patients

This study was a retrospective cohort study based on the existing clinical follow-up system, complied with the principles of the Declaration of Helsinki, and approved by the National Unit of Clinical Trial Ethics Committee, Peking University First Hospital. The data analyzed were of patients with PAC/PACG who were treated consecutively at the Peking University First Hospital Eye Department from January 2016 to May 2018. The diagnoses of PAC and PACG were based on the diagnostic criteria of the International Society of Geographic and Epidemiologic Ophthalmology (ISGEO) [12]. Operative indications included: 1. The degree of PAS>180° (This criterion was set according to Chinese Guidelines of Glaucoma Diagnosis and Treatment, which suggests that eyes with more than two quadrants of synechia angle closure need filtering surgery) [13]; 2. patients with high IOP or with normal IOP on at least one anti-glaucoma medication (normal IOP refers to IOP ≤ 21 mmHg); 3. The existence of cataract, and best corrected visual acuity (BCVA) of patients was less than 0.5 or the contrast sensitivity was lower preoperatively. Exclusion criteria included: 1. Patients who had undergone prior intraocular surgery (LPI, laser iridoplasty and anterior chamber paracentesis excluded); 2. patients with ocular disease known to affect anterior segment anatomy, such as ciliary body or iris cysts, trauma and use of topical drugs affecting iris configuration; 3. patients with a history of uveitis; 4. those with a long term use of topical or systemic glucocorticoid. Patients were divided into 2 groups, an acute group and a chronic group, depending on whether they had a history of an acute closure.

### Surgical procedure

**Fig. 1:**
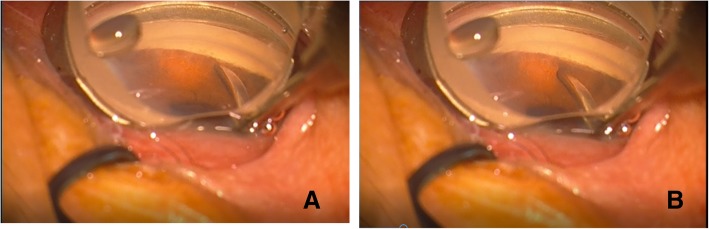
Images of the anterior chamber angle under gonioscopic visualization during Phaco-GSL. **a** The image of synechial angle-closure before Phaco-GSL. **b** The image of the reopened anterior chamber angle after Phaco-GSL at the same site. Phaco-GSL refers to phacoemulsification combined with goniosynechialysis

All surgeries were performed under topical anesthesia by one single surgeon (Li M). A clear corneal incision phacoemulsification was performed with an IOL implantation within the capsular bag. Viscoelastic then was injected in the anterior chamber, especially into the anterior angles, to substantially deepen the peripheral anterior chamber. The operating microscope was then tilted approximately 45° and a Swan Jacobs direct gonio-lens (Ocular Instruments, Bellvue, WA, USA) placed on the cornea to visualize the angle. Visible PAS was gently broken under direct visualization by pushing down on the peripheral iris with a flat spatula through the main or side incision. The end-point of the procedure was visibility of the scleral spur (Fig. 1). At a minimum, 240° (2:00~10:00) of angle could be seen and treated in this manner. The superior section was the most technically difficult to see and often was treated without the guidance of a gonio-lens. At the end of the procedure, the viscoelastic was replaced with balanced salt solution and incisions were hydrostatically sealed. Postoperatively, all patients received topical antibiotics for 1 week, as well as topical steroids, which were tapered over a period of 4 to 6 weeks depending on clinical need. Anti-glaucoma medications were discontinued postoperatively and restarted if needed.

### Examination

All patients underwent a baseline examination prior to surgery. Follow-up examinations were at 1 day, 7 days, 1 month and 3 months after the operation. Some of the patients completed longer follow-ups of every half year, beginning at 6 months after surgery. Routine ophthalmic examinations were done at each post-surgical follow-up visit. Gonioscopy was performed at every follow up visit beginning at the week one post-op exam. BCVA, visual field and ultrasound biomicroscopy (UBM) were performed 3 months after the operation. The follow-up visits and number of examination procedures were increased when deemed necessary.

The analysis index included: vision (Snellen visual chart); IOP (non-contact IOP measurement was taken on the 1st and 7th day after the operation, while Goldmann tonometer IOP measurement was taken at the other follow-ups); central anterior chamber depth (CACD), measured with the built-in measuring procedure of UBM; pupil size and activity; the degree of optic disc damage; intraoperative and postoperative complications, and PAS (°). The extent of glaucomatous optic neuropathy was classified by degree of optic nerve damage. No prominent damage: Vertical cup-to-disc ratios (VCDR) < 0.7 and VCDR asymmetry < 0.2, no focal thinning, notching of the neuroretinal rim; mild damage: VCDR≥0.7 or VCDR asymmetry≥0.2, or focal thinning, notching of the neuroretinal rim, but VCDR<0.8; advanced damage: VCDR≥0.8 [12, 14, 15].

For our study, surgical success referred to IOP ≤21 mmHg without any other treatment. While surgical failure was defined by two conditions: elevated IOP, including one single Goldmann tonometer measurement ≥25 mmHg, or at least two measurements ≥22 mmHg, excluding measurements affected by residual viscoelastic or glucocorticoids; or a large degree of re-PAS requiring intervention, that is the degree of re-PAS exceeded half the degree of PAS preoperatively from 2:00 to 10:00, and in the same time, the extent of PAS ≥ 180° in total.

### Statistical analyses

For comparisons between the acute and chronic groups, gender, visual acuity, pupillary dilation rate, complication rate, success rate and surgical failure rate, were analyzed with χ^2^-test (Fisher’s exact test) or Mann-Whitney Test. While age, IOP and the degree of angle synechiae were analyzed with Student’s t-test (independent samples test). For comparison of before and after surgical findings within the same group, Wilcoxon Signed Ranks Test was used. Probability values of less than 0.05 were considered statistically significant.

## Results

Twenty-nine consecutive PAC/PACG patients with cataract and extensive PAS were treated with Phaco-GSL. Two patients (one with a history of acute attack and another without) did not return for the 1 week post surgical follow-up visit. When follow-up telephone calls were made within 1 year after surgery, these patients expressed no discomfort and their IOP, performed in a nearby hospital, was normal. These 2 patients were not included in this study. There were 13 and 14 patients in the acute group and the chronic group respectively. The interval between acute attack and surgical intervention ranged from 14 days to 1 year (8 cases in 1 month, 2 cases in 1-3 months, 3 cases in 3 months-1 year). The general condition of the two groups before surgery is shown in Table 1. The incidence of pupil dilatation in the acute group was higher than that in the chronic group (p<0.05). While, the degree of optic disc damage of acute group was milder (p<0.05). There was no statistical difference in other aspects.

**Table 1:** The general condition of all subjects

	A group	C group	*P* value
Age (years)			
Mean ± SD	65.00 ± 9.54	65.14 ± 8.48	0.967^a^
Range	51~87	48~75	
Gender (%)			
Male	3 (23.1)	3 (21.4)	1.000^b^
Female	10 (76.9)	11 (78.6)	
Eye (%)			
right	6 (46.2)	4 (28.6)	0.440^b^
left	7 (53.8)	10 (71.4)	
Follow-up time (months)			
Mean ± SD	9.23 ± 6.53	9.00 ± 5.89	0.924^a^
Range	3~18	3~18	
Optic nerve damage			
No prominent damage	7 (53.8%)	2 (14.4%)	0.019^c^
mild	5 (38.5%)	6 (42.6%)	
advanced	1 (7.7%)	6 (42.6%)	
Dilated pupil (%)			
Yes	12 (92.3)	1 (7.1)	<0.001^b^
No	1 (7.7)	13 (92.9)	

Absolute values (percentage), unless stated otherwise. *SD* Standard deviation

^a^Student’s t-test (independent samples test)

^b^χ^2^-test (Fisher’s exact test)

^c^Mann–Whitney U-test

The pre-operative visual acuity of the acute group was lower than that of the chronic group, the difference of which was statistically significant. There was no significant difference in visual acuity between the two groups at the 3 month follow-up visit (Table 2).

**Table 2:** Visual acuity in two groups before and after the operation

	Acute group(n)					Chronic group(n)					Z value	*P* value
	0	0~<0.1	0.1~0.25	0.3~0.5	≥0.5	0	0~<0.1	0.1~0.25	0.3~0.5	≥0.5		
baseline	0	8	3	1	1	1	0	2	4	7	3.08	0.002^a^
Postoperative 3 m (UCVA)	0	0	1	8	4	1	0	0	5	8	1.19	0.302^a^
Postoperative 3 m(BCVA)	0	0	1	1	11	1	0	0	1	12	0.039	0.981^a^

^a^Mann–Whitney U-test. *UCVA* Uncorrected visual acuity, *BCVA* Best corrected visual acuity

For all the patients, visual acuity post-operatively was better than that pre-operatively, with the exception of one patient in the chronic group, whose visual acuity was no light perception before surgery. Goldmann perimetry was used to assess visual fields of some patients because they were unable to cooperate with automated perimetry. Therefore, the results of perimetry were not analyzed in our study.

Pre-operative and post-operative IOP, ACD, the range of PAS and the need for medication in the two groups are shown in Table 3. After surgery, the IOP decreased, the need for topical medication was reduced and the anterior chamber depth deepened in both groups. The need for systemic medication decreased in the acute group. All these differences were statistically significant. There was no significant difference between the two groups except for a shallower CACD and greater systemic medication use preoperatively in acute group.

**Table 3:** Comparison of IOP, the need for topical and systemic medications, CACD and the degree of PAS between the two groups prior to and after surgical intervention

	Acute		chronic		*P* value^b^
		*P* value^a^		*P* value^a^	
IOP (mmHg, mean ± SD)					
Baseline	29.77 ± 11.55		26.00 ± 11.2		0.398^a^
Postoperative 1 m	15 ± 1.63	0.003^b^	17 ± 4.31	0.022^b^	0.105^a^
Postoperative 3 m	14.92 ± 1.66	0.003^b^	14.93 ± 2.7	0.001^b^	0.995^a^
Number of drops					
Baseline					
Median (min~max)	3 (2~4)		3 (1~4)		0.202^c^
1	0 (0%)		1 (7.1%)		
2	2 (15.4%)		5 (35.7%)		
3	8 (61.5%)		6 (42.9%)		
4	3 (23.1%)		2 (14.3%)		
post-op 3 m					
Median (min~max)	0 (0~0)	0.001^b^	0 (0~2)	0.002^b^	
0	13 (100%)		10 (71.4%)		0.220^c^
1	0 (0%)		1 (7.1%)		
2	0 (0%)		3 (21.4%)		
Number of systemic drugs					
Baseline					
Median (min~max)	1 (0~2)		0 (0~1)		0.001^c^
0	3 (23.1%)		13 (92.9%)		
1	9 (69.2%)		1 (7.1%)		
2	1 (7.7%)		0 (0%)		
Postoperative 3 m					
0	13 (100%)	0.002^b^	14 (100%)	0.317^b^	–
CACD (mm)					
Baseline	1.68 ± 0.27		1.89 ± 0.23		0.042^a^
Postoperative 3 m	3.29 ± 0.22	0.001^b^	3.40 ± 0.25	0.001^b^	0.226^a^
PAS (°, mean ± SD)					
pre-op	314.23 ± 49.07		285.00 ± 53.28		0.152^a^
post-op 3 m	116.54 ± 73.78	<0.001^b^	156.43 ± 56.35	<0.001^b^	0.125^a^

^a^Postoperative versus baseline; ^b^ acute group versus chronic group. *SD* Standard deviation, *IOP* Intraocular pressure, *CACD* Central anterior chamber depth, *PAS* Peripheral anterior synechiae

^a^Student’s t-test (independent samples test)

^b^Wilcoxon Signed Ranks Test

^c^Mann–Whitney U-test

Because of poor corneal transparency or poor patient cooperation, in 3 cases of the acute group and in 2 cases of the chronic group, angle separation was completed without the guidance of a gonio-lens; in all these cases, both the IOP and the need for medication improved significantly. Under gonioscopy, 1 week after surgery, the angle was observed to open more than 180° in 1 case of the chronic group, less than 180° in 1 case of the chronic group and in 3 cases of the acute group.

At the 3 month post-operative follow-up visit, all patients in the acute group and 9 patients in the chronic group no longer needed any medical treatment, including anti-glaucoma medication. The success rates for the procedure were 100 and 64.3% for the acute and chronic groups respectively (*p* = 0.041). In the chronic group, 5 cases failed, with 3 cases still needing topical medication to control IOP and 2 cases receiving LPI treatment because of extensive re-PAS at 1 week after the operation. There were 3 cases (30%) in the acute group and 10 cases (83.3%) in the chronic group with varying degrees of re-PAS within 3 months after the operation. This difference was statistically significant (*p* = 0.01).

The success rate of surgery, bleeding and pain during the operation, fibrinous reaction in anterior chamber and re-PAS post-operation for both groups are shown in Table 4. During the operation, 1 patient in the acute group suffered from a partial rupture of an inferior lens suspensory ligament. Since the extent of the rupture was not very extensive, it was not dealt with during the operation. The IOL did move up slightly and BCVA was 0.7 postoperatively.

**Table 4:** Comparison of complications and surgical failure rate post-operation

	Acute	chronic	*P* value
Pain (number,%)	6 (46.2%)	3 (21.4%)	0.236^a^
Hemorrhage (number,%)	8 (61.5%)	8 (57.1%)	1.000^a^
Fibrinous reaction (number,%)	6 (46.2%)	0 (0%)	0.006^a^
Failure (number,%)	0 (0%)	5 (35.7%)	0.041^a^
Re-PAS (number,%)	3 (30%)	10 (83.3%)	0.011^a^

Absolute values (percentage)

^a^Chi-Square Tests (Fisher’s Exact Test)

*Re-PAS* Recurrence of peripheral anterior synechiae

The occurrence time of re-PAS in the two groups is shown in Table 5. In the acute group, re-PAS in all 3 cases occurred within 1 week after the operation, and did not progress further. For the chronic group, re-PAS in 7 cases occurred within 1 week of surgery. Among these 7 cases, in 3 cases, re-PAS did not progress beyond the 1 week follow-up and in 3 cases re-PAS continued to progress for 1 month after the operation (follow-up times were 3, 6, 18 months respectively). In the remaining 1 case, the process of re-PAS continued for 3 months after surgery (follow-up period was 12 months). In the 3 other cases of the chronic group, the re-PAS appeared at 1 month post-operatively with no further changes. (The follow-up period was more than 12 months). None of the patients who were followed for more than 3 months had new re-PAS beyond the 3 month follow-up visit.

**Table 5:** The occurrence time of re-PAS postoperatively (+: yes, −: no)

	Case number	≤1 W	1 W~1Mon	1Mon~3Mon
Acute	3	+	–	–
	7	–	–	–
Chronic	1	+	+	+
	3	+	+	–
	3	+	–	–
	3	–	+	–
	2	–	–	–

The degree of re-PAS was small (<90°) in 8 cases (2 cases in the acute group and 6 cases in the chronic group) and large (≥90°) in 5 cases (1 case in the acute group and 4 cases in the chronic group). In the cases with re-PAS more than 90°, for all 5 cases, re-PAS appeared 1 week after the procedure. In 2 of the chronic cases, at the 1 week follow-up, both the central and peripheral anterior chambers were shallow. The possibility of malignant glaucoma was considered in these cases. Topical anti-glaucoma medication, mydriasis treatment and enhanced steroid medication were then given. As a result, the central anterior chambers deepened, but the extent of PAS was similar to the pre-surgical presentation. Because laser iridoplasty failed to re-open the angle at 1 month after surgery, anti-glaucoma drugs were administered to maintain normal IOP. In the other 2 chronic cases and 1 acute case, the central anterior chamber was moderately deep while the peripheral chamber was shallow at 1 week follow up. Because the degree of re-PAS exceeded half the degree of PAS preoperatively from 2:00 to 10:00 in the two chronic cases, laser iridoplasty was performed immediately, and as a result, most of the angle re-opened. In all patients, the sites of re-PAS were identical to the preoperative PAS sites and did not extend beyond the preoperative PAS range.

With the exception of 3 patients with CPACG, who were already being treated with medication, at follow-up visits beyond the 3 months post-surgical mark, no patient IOP was ever greater than 21 mmHg. However, topical drugs were prescribed to get lower IOP in 1 APACG patient and in 3 CPACG patients who had serious optic nerve damage. No further PAS change was observed in any of the patients.

## Discussion

This study analyzed the clinical effects of Phaco-GSL on PAC/PACG patients with extensive PAS. Differences in effect were noted between the acute and the chronic patient groups. Although the IOP and the need for medical treatment decreased in both groups, in the chronic group, both the surgical failure rate and the incidence of re-PAS after the surgery were significantly higher than in acute group (*p* < 0.05). Meanwhile, we also observed that re-PAS occurred primarily within 1 month of surgery and all the re-PAS greater than 90°occurred within 1 week. To the best of our knowledge, this is the first study to document the precise time of re-PAS after Phaco-GSL surgery in Chinese patients with PAC/PACG.

Maintaining an adequately open angle was critical for IOP normalization in PAC/PACG patients. In this study both the surgical failure rate and the re-PAS rate in the chronic group were higher than in the acute group (chronic group vs acute group: 35.7% vs 0 and 83.3% vs 30%, respectively), suggesting that re-PAS was more likely to occur in patients in this group, and that they were more likely to require medication or other interventions after surgery. There has been limited discussion in the literature in regard to the post-surgical differences in effect between acute and chronic PAC/PACG after Phaco-GSL treatment. Kameda T and coauthors [7] retrospectively analyzed the related factors of failure after Phaco-GSL in 109 patients with PAC/PACG, showing that history of an attack was not a protective factor. However, the authors admitted that the cases that they analyzed retrospectively were from 5 different hospitals over a 10-year period, and that there might have been deviations both in case selection and in operative technique. Moreover, the cases they included required only more than 90° PAS, which was different from our inclusion criterion for PAS (more than 180°). In terms of mechanism, pupil block was the main cause of PAS in acute patients, while for chronic patients, usually multiple factors were involved [10]. Although there were studies showing that phacoemulsification relieved not only pupil block, but also attenuated anterior positioning of the ciliary process in eyes with PAC [16], other papers reported that patients with a shallow angle with plateau iris did not significantly improved after cataract surgery [17]. Therefore, the shallow condition of the peripheral anterior chamber could have persisted after Phaco surgery in some chronic patients. And, if the angle were narrow enough, post-surgical inflammation would be an additional factor that might lead to re-closure of the newly re-opened angle. Additionally, malignant glaucoma is more likely to occur in patients with chronic PAC/PACG [18]. This form of glaucoma can be induced by ciliary body hypertrophy resulting from postoperative inflammation, leading to widespread shallowing of the anterior chamber and secondary angle closure. In this study, all patients with greater than 90° re-PAS had a shallow anterior chamber postoperatively, 3 cases with only a shallow peripheral anterior chamber, and 2 other cases with both peripheral and central shallow anterior chamber. Because the number of cases was small, we did not analyze further any other risk factors for re-PAS.

The time at which re-PAS occurred after the procedure was an important outcome of this study. During the operation, the angle of patients from 2:00 to 10:00 was separated precisely under the guidance of gonio-lens. Therefore, the presence of PAS in this area after surgery could be considered as an occurrence of re-PAS. Many researchers did not evaluate the angle condition after surgery [7, 19], or observed it only 2 to 3 months after surgery [4–6, 11]. In our study, regular anterior chamber angle measurements were performed beginning at 1 week after surgery. It was observed that re-PAS formed very early post-operatively and that all large-scale re-PAS (≥90°) occurred within 1 week after surgery. At this early post-surgical time-point, laser iridoplasty is efficacious for patients whose anterior chamber is shallow only in the periphery. For patients whose anterior chamber is shallow both centrally and peripherally, administration of drugs to induce ciliary body paralysis and use of more potent anti-inflammation drugs are more critical. Our results also showed that there was no significant change in the degree of angle opening after the first 3 month postoperative period. These findings suggest that it is incumbent upon the surgeon to check for re-PAS during the early post surgical period in patients on whom Phaco-GSL has been performed. This is especially important for patients with chronic closures. In order to assess the extent of re-PAS, gonioscopy should be performed on patients with elevated IOPs or shallow anterior chamber early in the postoperative period, and extensive re-PAS then could be found and treated in a timely manner.

This study had several limitations, such as the small number of patients, the relatively short follow-up time, a possible bias in angle evaluation, and some differences in features between the two groups. However, we observed the wide range of re-PASs occurring only within 1 week after surgery. Also, we found no new PAS appearing in patients who were followed for more than 3 months. Teekhasaenee C and coauthors [4] followed patients with a history of acute attack who were treated by Phaco-GSL. These patients were examined every 3 months, initially at 3 months after surgery, and they found that the success rate did not change postoperatively past 3 months, and that the angle condition remained stable for 6 years. Therefore, the effect on the results in our study of having a minimum follow-up of 3 months may be minimal or insignificant. Also, because of the difficulty of PAS assessment in patients with a shallow angle, there may have been deviations in the gonioscopic examination of the PAC/PACG patients before surgery. This evaluation was easier and more accurate after surgery because the anterior chamber had deepened. So, the effect of this factor on the post-operative re-PAS results of our study might be minor. We did note several differences in preoperative status and in complications between the two groups. For example, patients in the acute group had larger pupils, shallower anterior chambers and a higher incidence of postoperative anterior chamber fibrinous reaction. These factors might have affected the rate of re-PAS in our study. However, these factors were likely to increase the incidence of PAS. Even though the risk factors of re-PAS were higher in the acute group, the re-PAS rate was still lower than that of the chronic group, suggesting that these factors were not contributory to our results.

In future studies we intend to increase the number of cases to verify any difference between these groups. Also, in the future, other examination methods, such as those capable of assessing more objectively and precisely the anterior chamber condition within 1 week after surgery, would be performed to clarify further the risk factors of re-PAS after surgery. These efforts may assist us in providing more detailed information in regard to the post-operative treatment and management of PAC/PACG patients.

In summary, despite the limitations of the study, combined phacoemulsification with goniosynechialysis appears to be an effective and safe treatment in the control of IOP and reduces the need for medication in patients with both acute and chronic PAC/PACG. However, in chronic patients, there is a greater likelihood of re-PAS after surgery, for some of which additional treatments were needed. Our results suggest that large degree re-PAS following Phaco-GSL often occurs within 1 week postoperatively, indicating that patients need to be monitored closely during the early post-operative period. This is particularly true for those patients with chronic PAC/PACG.

## Abbreviations

BCVA: Best corrected visual acuity
CACD: Central anterior chamber depth
IOP: Intraocular pressure
LPI: Laser peripheral iridotomy
PAC: Primary angle closure
PACG: Primary angle closure glaucoma
PAS: Peripheral anterior synechiae
Phaco-GSL: Phacoemulsification plus goniosynechialysis
re-PAS: Recurrence of PAS
UBM: Ultrasound biomicroscopy
VCDR: Vertical cup-to-disc ratios
